# Development of a Mercury Bromide Birefringence Measurement System Based on Brewster’s Angle

**DOI:** 10.3390/s23094208

**Published:** 2023-04-23

**Authors:** Oh-Tae Kwon, Geonwoo Kim, Hyungjin Bae, Jaeyeol Ryu, Sikwan Woo, Byoung-Kwan Cho

**Affiliations:** 1 Department of Biosystem Machinery Engineering, College of Agricultural and Life Science, Chungnam National University, 99 Daehak-ro, Yuseong-gu, Daejeon 34134, Republic of Korea; got8054@naver.com (O.-T.K.); snowballgame@o.cnu.ac.kr (H.B.); 2 Department of Biosystem Bio-Industrial Machinery Engineering, College of Agricultural and Life Science, Gyeongsang National University, 501 Jinju-daero, Jinju 52828, Republic of Korea; 3 Institute of Agriculture and Life Science, Gyeongsang National University, Jinju 52828, Republic of Korea; 4 Department of Space Optics R&D, Green Optics, 45, Ganri 1-gil, Cheongwon-gu, Cheongju 28126, Republic of Korea; jyryu@greenoptics.com (J.R.); sgwoo@greenoptics.com (S.W.); 5 Department of Smart Agricultural System, College of Agricultural and Life Science, Chungnam National University, 99 Daehak-ro, Yuseong-gu, Daejeon 34134, Republic of Korea

**Keywords:** AOTF, Hg_2_Br_2_ crystal, birefringence measurement, Brewster’s angle

## Abstract

Mercury bromide (Hg_2_Br_2_) has been used to develop acousto-optic tunable filters (AOTFs) because it has several advantages, including a high refractive index, a broad optical bandwidth, and a relatively high figure of merit. Therefore, the measurement of its birefringence is a highly important factor for ensuring AOTF quality. However, for single crystals, it is difficult (at the millimeter scale) to quantify the birefringence using an ellipsometer, as this equipment is only designed to conduct measurements on thin films. In this study, a simple birefringence measurement system for Hg_2_Br_2_ was developed based on Brewster’s angle at the millimeter scale. The planar distributions of the Hg_2_Br_2_ crystal along the (100), (010), and (001) planes were used in the experiments. The developed measurement system can measure the reflected light intensity of the Hg_2_Br_2_ crystal depending on the incidence angles (rotations at 0.01125° steps) and can calculate the ordinary and extraordinary refractive indices and birefringence. The calculated birefringence of the Hg_2_Br_2_ crystal was 0.8548; this value exhibits an error of 0.64% compared with a value of 0.86 reported in the literature. The developed measurement system demonstrates the ability to be used to evaluate the quality of birefringent single crystals.

## 1. Introduction

An acousto-optic tunable filter (AOTF) is a highly integrated device that combines optical and acoustic components to achieve wavelength-dependent filtering and tunability. AOTFs work by producing a traveling acoustic wave in a crystal, which in turn creates a spatially varying birefringence in the crystal [1]. This birefringence allows the crystal to act as a wavelength-dependent diffraction grating, deflecting the light of a specific wavelength in a particular direction. The deflection angle is dependent on the wavelength of the input light and the frequency of the acoustic wave. By changing the frequency of the acoustic wave, the wavelength of the diffracted light can be tuned [2].

Accordingly, it has also been used to develop hyperspectral imaging (HSI) systems to selectively filter out specific wavelengths of light [3]. An HSI system can generate a sample image by utilizing an AOTF to produce light of specific wavelengths. Furthermore, the integration of AOTFs into HSI systems offers several benefits, such as compactness, high resolution, and faster imaging speeds, all without the need for mechanical moving parts [4]. By selectively filtering out unwanted wavelengths, the resultant HSI images can provide detailed spectral information about the sample, which can be helpful for a wide range of applications, such as agricultural applications [5], remote sensing [6], environmental monitoring [7], and medical imaging [6,8]. 

The main components of an AOTF include a crystal, an ultrasonic transducer, and an input/output optical system. As noted above, the crystal is typically made of a birefringent material, such as tellurium dioxide (TeO_2_) or quartz [2,7]. The crystal is designed to have a specific orientation and thickness to optimize the interaction between the acoustic and optical waves. It facilitates the wavelength-dependent diffraction of light, which is the basis of AOTF operations [4]. Then, the amount of birefringence in the crystal determines the frequency range over which the AOTF can operate and the resolution of the HSI device. The birefringence also affects the efficiency of the AOTFs and the amount of power that can be transmitted through the crystal [9,10]. Therefore, the accurate measurement of the crystal birefringence is critical in the design and optimization of AOTFs.

Among the various crystals for AOTF applications, mercury bromide (Hg_2_Br_2_) has been widely used because of its high refractive index and high acousto-optic figure of merit, which is a measure of how efficiently a material can convert an acoustic wave into an optical wave [2,11]. Another advantage of mercury bromide is that it has a relatively wide acoustic bandwidth (0.4 to 30 µm), that allows for a wide tuning range in AOTFs. It also allows for the filtering of a wide range of wavelengths in the optical signal [12,13].

In recent times, ellipsometers have been widely used to measure birefringence, that quantifies the differences in the light phase using 45° polarization and the retardation of the light phase using the quarter-waveplate. The ellipsometer measures the amplitude ratio (ψ) and phase difference (Δ) between p- and s-polarized light waves [14]. Once these values have been measured, it is possible to calculate the refractive index based on the ellipsometric ratio (ρ). Hence, the refractive index can be calculated by measuring the amplitude ratio and phase difference between p- and s-polarized light waves [15].

However, ellipsometers encounter problems in the quality tests of birefringent crystals. The ellipsometer was designed to conduct birefringence measurements in thin films [16,17,18]. It is thus unsuitable for the measurement of birefringent crystals at the millimeter scale—in conjunction with the use of the AOTF—as opposed to the intended nanometer-scale of thin-film measurements [18]. Moreover, the accurate interpretation of ellipsometric data requires a good understanding of the sample’s crystal structure, orientation, and other properties that can affect the polarization of light passing through the sample [19,20,21]. Therefore, careful experimental design and data analysis are required to accurately measure the birefringence of mercury bromide using ellipsometry.

Herein, to overcome the above limitations, a simple birefringence measurement system for a single crystal was investigated using the Brewster angle. As noted above, birefringence can be measured by analyzing the change in polarization of the reflected or transmitted light. The light is typically polarized in a specific direction, such as in p-polarization (parallel to the plane of incidence) or s-polarization (perpendicular to the plane of incidence). As the polarized light passes through the AOTF and interacts with the two orthogonally polarized components, the polarization state of the light changes, resulting in a change in the phase difference between the two components. The features of the Brewster angle are twofold: (a) the angle between the reflected and refracted light is 90° and (b) the incidence of p-polarized light yields reflected light that is not p-polarized. Therefore, we demonstrated that it was possible to identify the Brewster angle, such that the intensity of p-polarized light is zero by measuring the p-polarized light intensity and deriving each refractive index of the Hg_2_Br_2_ crystal.

## 2. Materials and Methods

### 2.1. TeO_2_ Single Crystal

**Figure 1:**
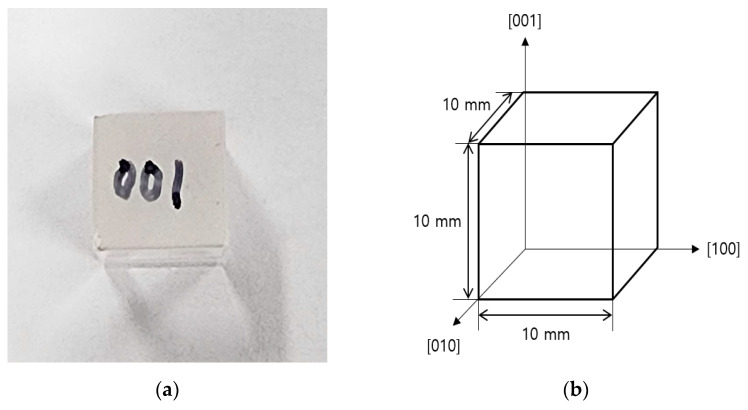
Information on the three planes of the TeO_2_ crystal used in the experiment. (**a**) The TeO_2_ crystal used in experiment., (**b**) Information about the TeO_2_ crystal.

Prior to measuring the birefringence of a Hg_2_Br_2_ crystal using the Brewster angle, the birefringence of a tellurium dioxide (TeO_2_) crystal (CryLink, Nanjing, China), which is commonly used in AOTFs [22], was measured for validation of the developed measurement system. TeO_2_ crystals used in the current study are transparent from visible- to mid-wave infrared regions (0.33~5.0 μm). It was cut into cubic structures that included the (100), (010), and (001) planes to facilitate birefringence measurements. The TeO_2_ crystal length in [100], [010], and [001] directions was 10 mm, and the surface of the [100] plane was polished for measuring the reflected light intensity (Figure 1).

### 2.2. Hg_2_Br_2_ Single Crystal

Hg_2_Br_2_ crystals are transparent in the range between the visible and long wave infrared region. The Hg_2_Br_2_ crystal used in this study (Green Optics, Cheongju, Republic of Korea), was fabricated using the physical vapor transport method (PVT), and its birefringence was measured. It is necessary to investigate the complete process in a cautious and organized manner, as the formation of atomic and bulk defects in PVT-based Hg_2_Br_2_ crystals is significantly influenced by the material’s purity and the crystal growth conditions. To achieve this goal, three primary procedures were carried out: purification, growth of crystals, and assessment of quality.

Prior to crystal growth, the Hg_2_Br_2_ powders were purified through the PVT process. The purification involved cleaning the quartz ampoule with a series of wet chemicals, including high-purity acetone, isopropyl alcohol, and ultrapure water, to remove impurities. The ampoule was then dried at 150 °C for an hour at a pressure of 10^−3^ torr to eliminate any remaining moisture inside. Next, 4–9 grade (99.99%) Hg_2_Br_2_ powder was placed into the ampoule and dried again at 100 °C for an hour at 10^−6^ torr to evaporate any unintentional residual moisture. Finally, the Hg_2_Br_2_ raw powder underwent purification at 300 °C for 10 h under high vacuum conditions of 10^−6^ torr, with the chamber slowly cooled at a rate of 1 °C/min to prevent the nucleation of Hg atoms and related secondary phases. As a result of this process, a highly purified Hg_2_Br_2_ powder was acquired.

Following the purification process, the Hg_2_Br_2_ powder was placed in a quartz ampoule and a high vacuum of 10^−6^ torr was established. A temperature gradient profile was set up between two (lower and upper) heaters to allow the Hg_2_Br_2_ raw powder to sublimate, and the sublimated sources were transferred to the growth zone. The sublimated powder was condensed in the growth zone, and a Hg_2_Br_2_ crystal seed was produced in the growth zone on the ampoule bottom. The crystals then grew from the seed at a rate of 0.35 mm a day, with the temperature gradient of the ampoule optimized for growth. Then, the crystal was slowly cooled to prevent cracks caused by shrinking.

**Figure 2:**
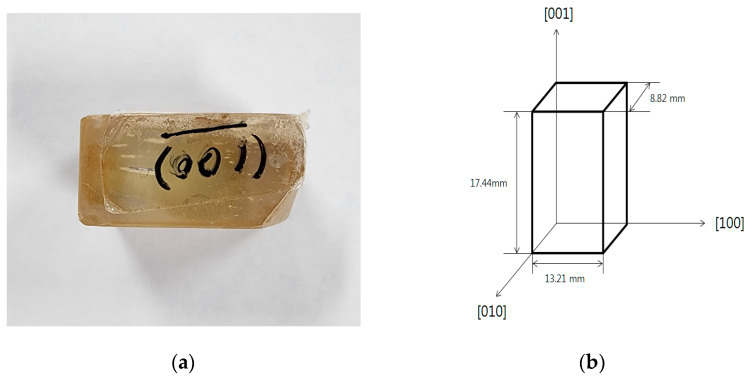
Information on the three planes of the Hg_2_Br_2_ crystal used in the experiment. (**a**) The Hg_2_Br_2_ crystal used in the experiment. (**b**) Information about the Hg_2_Br_2_ crystal.

The quality of the fabricated Hg_2_Br_2_ crystal was evaluated using Raman spectroscopy and transmission electron microscopy (TEM), and its atomic vibration mode, crystallinity, and structure were identified. In our previous study, the X-ray diffraction (XRD) result of Hg_2_Br_2_ powder was shown, and the raw and standard data of (110) and (220) planes were also investigated [23]. Others peaks of (101), (004), and (204) were also clearly discovered. Following the growth of the Hg_2_Br_2_ crystal, major XRD peaks on (110) and (220) planes were defined. In addition, significant Raman peaks of Hg_2_Br_2_ were observed at 35.5, 91, 136, and 221 cm^−1^ [23]. Following the characterization of the Hg_2_Br_2_ crystal, it was cut into cuboidal structures that included the (100), (010), and (001) planes to facilitate the birefringence measurements (Figure 2a). The Hg_2_Br_2_ crystal lengths along the [100], [010], and [001] directions were measured as 13.21 mm, 8.82 mm, and 17.44 mm, as shown in Figure 2b. Following the cutting process, the plane (100) was made transparent by polishing it to measure the birefringence.

### 2.3. TeO_2_ and Hg_2_Br_2_ Crystal Index Ellipsoid

The refractive index derivation based on the Brewster angle measures the reflected light intensity of p-polarized light to derive the refractive index; thus, only one refractive index can be derived for each direction on each surface [21]. Therefore, for a single birefringent crystal with multiple refractive indices, it is necessary to determine the refractive index distribution along the plane direction of the single crystal to measure the Brewster angle and calculate each refractive index. The index ellipsoid represents the distribution of the refractive index according to the polarization direction of light [14]. The refractive index can be changed according to the polarization direction of the incident light and the direction of the incident surface. The refractive index was measured considering the index ellipsoid.

**Figure 3:**
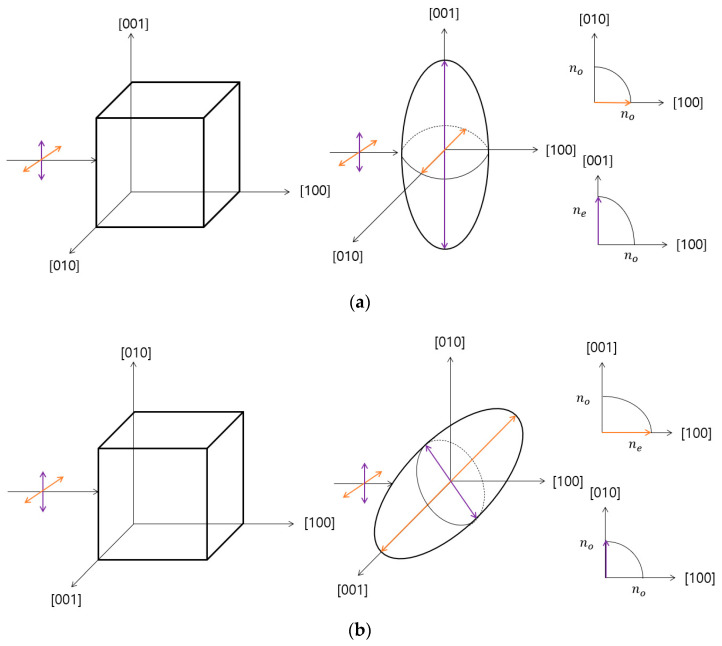
Index ellipsoid of the Hg_2_Br_2_ and TeO_2_ crystals in different planar directions. (**a**) Index ellipsoid of the Hg_2_Br_2_ and TeO_2_ crystals’ refractive index for which the *z*-axis was [001]. (**b**) Index ellipsoid of the Hg_2_Br_2_ and TeO_2_ crystals’ refractive index for the 90° rotation with respect to [100].

The Hg_2_Br_2_ and TeO_2_ crystals are uniaxial with two refractive indices; they have an ellipsoidal shape in which the extraordinary refractive index (n_e_) is larger than the ordinary refractive index (n_o_) [14,24,25]. In the single uniaxial crystal, only one refractive index appears when light impinges on a specific surface that has the same optical axis direction. The Hg_2_Br_2_ and TeO_2_ crystal has an optical axis of (001) and the birefringence does not occur according to the direction of the s- and p-polarized light [24,26]. Therefore, to measure the two refractive indices of Hg_2_Br_2_ and TeO_2_ crystals considering the index ellipsoid, the (100) plane is irradiated for obtaining the different refractive indices. The location directions of Hg_2_Br_2_ and TeO_2_ crystals are shown in Figure 3 for irradiating p-polarized light on the perpendicular (100) plane. The refractive index of the polarized light following laser irradiation on the (100) plane is shown in Figure 3. Figure 3a shows that, when light is irradiated on the (100) plane, p-polarized light has a refractive index denoted by n_o_, and s-polarized light has a refractive index denoted by n_e_. Additionally, when rotated 90° based on the (100) plane in Figure 3b, p-polarized light has a refractive index of n_e_, and s-polarized light has a refractive index of n_o_. Therefore, it is essential to fix the irradiating plane and rotate the crystal 90° to derive the birefringence using the Brewster angle. 

### 2.4. Measurement of the Brewster Angle

#### 2.4.1. Brewster Angle

**Figure 4:**
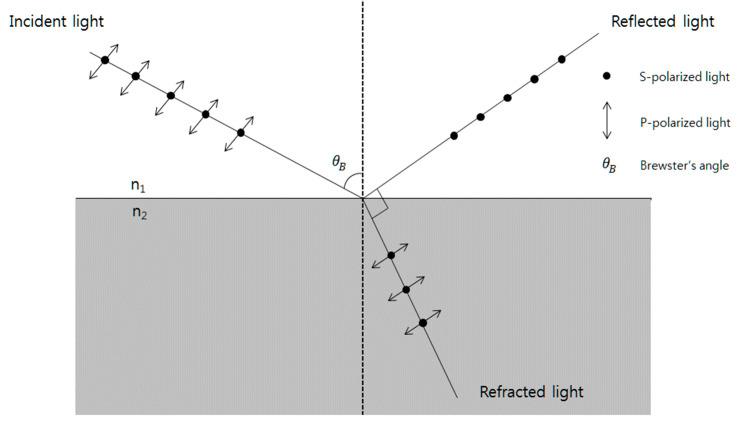
Brewster angle schematic.

The Brewster angle’s features are twofold in Figure 4: (a) the angle between the reflected and refracted light is 90° and (b) the incidence of p-polarized light yields reflected light that is not p-polarized. Moreover, the Brewster angle is related to the refractive index according to Equation (1). It is possible to identify the Brewster angle, such that the intensity of p-polarized light is zero by measuring the p-polarized light intensity and deriving each refractive index of the Hg_2_Br_2_ and TeO_2_ crystals.

The Brewster angle is determined by measuring the intensity of reflected light when p-polarized light reaches zero intensity; it was accomplished by rotating the crystals. Therefore, the measurement system can control the incidence angle of the light irradiated on the Hg_2_Br_2_ and TeO_2_ crystals and can derive the refractive index by Equation (1). The control method for measuring reflected light is the rotation of each crystal and detector of intensity.

(1) $$n_{2}=n_{1}\;\tan\;\theta_{B},$$

where *n*_1_, *n*_2_, and *θ_B_* are the refractive indices of media 1 and media 2, and the Brewster angle, respectively.

#### 2.4.2. Configuration of the Measurement System

To measure the precise birefringence of the Hg_2_Br_2_ and TeO_2_ crystals, a high-resolution system was developed. The developed system included a laser source, light intensity detector, rotational parts of the Hg_2_Br_2_ crystal and detector, and data acquisition parts of the light intensity module. The laser source used was a 5 mW, 632.8 nm, He-Ne laser (HNL050LB, Thorlabs Inc., Newtown, NJ, USA) with linear polarization at a 500:1 ratio. The He-Ne laser was attached to a height lab jack (L490/M, Thorlabs Inc., Newtown, NJ, USA) with a base plate for the appropriate irradiation of light to the crystal. The detector (S120C, Thorlabs Inc., Newtown, NJ, USA) used in the system could collect light in the wavelength range of 400–1100 nm and light intensities in the range of 50 nW–50 mW. The rotational parts comprised of two motors (EzM-60 Series, Fastech, Bucheon, Republic of Korea) and a motor drive (EzS-NDR-60L, Fastech, Bucheon, Republic of Korea) that had a resolution of 32,000 pulses per rotation (ppr). The drive provides a connection to a personal computer (PC) that controls the resolution of the rotation, as well as the rotational direction and range. The rotational parts are supported by a frame used to fix the motor and drive. The motors are combined with the sample stage (used for the rotation of the crystals) and the arm to achieve the detection of the reflected light.

**Figure 5:**
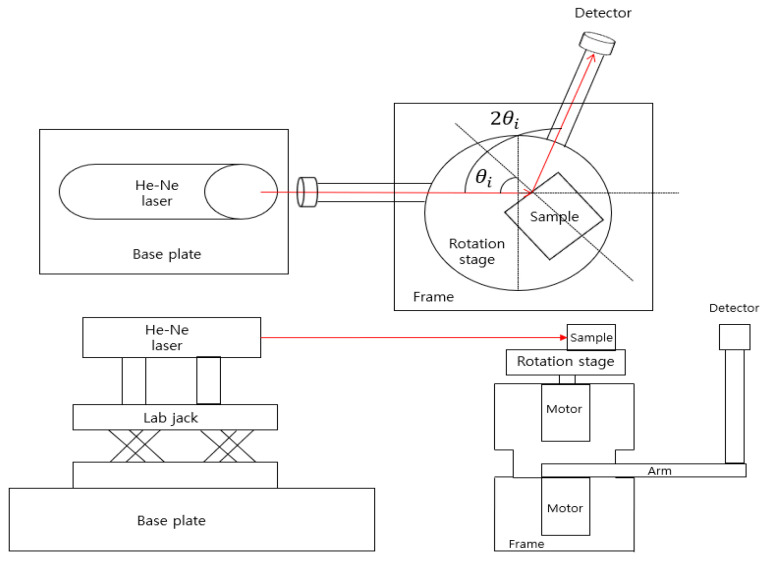
Schematic of the developed Brewster angle measurement system.

The developed system was set up (sequence of He-Ne laser and rotational parts with the detector), as shown in Figure 5. First, the base plate was fixed on the optical table and the He-Ne laser was mounted on the lab jack using an optical post (TR75/M, Thorlabs Inc., Newtown, NJ, USA) and post cage (HCM2/M, Thorlabs Inc., Newtown, NJ, USA). The frame was designed for combining the motor, drive, and arm. The frame parts of the coupled arm had sufficient space to be able to rotate over 180°. Additionally, the frame parts of the coupled motor accounted for the height to prevent vibration in case the motor contacted the frame. Furthermore, the rear part of the frame had an adjustable square space to fix the drive that connected the motor. The arm had an oval-shaped hole to reduce its weight and allow for the attachment of the detector’s rod. The detector was connected to the optical power meter (PM100D, Thorlabs Inc., Newtown, NJ, USA) for light intensity measurements. An RS-485 communication was used to control the motor. The light source element had a basic frame and a laser holder to fix the He-Ne laser. It was thus possible to match the center of the target crystal using the lab jack, which controlled the height (at the mm range) of the frame combined with the He-Ne laser. 

#### 2.4.3. Birefringence Measurements of TeO_2_ and Hg_2_Br_2_ Crystals

To measure the birefringence TeO_2_ and Hg_2_Br_2_ crystals, which is defined as the difference between n_o_ and n_e_, the reflected light intensity was measured to derive the n_o_ and n_e_. First, the He-Ne laser was warmed up for 15 min to attain a constant light intensity, as assessed by the detector. Subsequently, the p-polarization of the He-Ne laser was confirmed by the intensity of the linear polarizer, which was measured by the detector. Hg_2_Br_2_ was then aligned between the plane (100) of the Hg_2_Br_2_ crystal and the motor’s rotational axis. The motor’s resolution was then set to 0.01125°/pulse. The arm for handling the detector’s angle was also aligned with the 0° based on the line of the laser using the lab jack, and the first position was saved using a software program (Ezi-Plus R V6, Fastech, Bucheon, Republic of Korea). Regarding the reflective light intensity, when the TeO_2_ and Hg_2_Br_2_ crystals and rotation stage were rotated 0.01125°, the detector with the arm was rotated 0.0225°, as shown in Figure 5. The limited measurement range was precluded between the detector and the laser beam because the installed rear diameter of the detector was covered by the laser beam. Additionally, the measurement system prevented the angle range from reducing the measurement time; accordingly, the measurements were performed by specifying the range. Datasets of the TeO_2_ crystal were 51.3° to 78.9075° for the rapidly measured refractive index. Upon measuring the TeO_2_ crystal, datasets of Hg_2_Br_2_ crystal 5376 were obtained by measuring the reflected p-polarized light intensity from 24.6375° to 85.1175° in order to reduce the error rate of the refractive index. The intensity of the reflected light for the obtained angle of incidence was recorded 10 times, and the average value was used to reduce the intensity error of the He-Ne laser. P-polarized light can be measured when the reflected light is equal to zero along the direction of the index ellipsoid. Therefore, to obtain the Brewster angle in a different direction, each crystal was rotated 90° with respect to the (100) plane based on considerations of the index ellipsoid; this process was repeated to obtain the reflected light data.

Using the measured data, a regression graph was created in MATLAB (version 9.70 2019b, MathWorks, Natick, MA, USA) and the Brewster angle was derived by extracting the minimum point of the reflected light intensity in the graph obtained through regression. To obtain the point at which the intensity of reflected light is minimized owing to the surrounding environment, the environment light intensity was measured when the He-Ne laser was turned off. Following the elimination of noise intensity, the refractive index using Equation (1) related to the Brewster’s angle and the birefringence of the TeO_2_ and Hg_2_Br_2_ crystals were calculated.

## 3. Results

The reflected light intensity decreased as the angle changed from its first value to the Brewster angle on the crystals. Moreover, the reflected light intensity was increased when the angle changed from the Brewster angle to the final value. The reflected light intensity plot was consistent with the features of typical reflected light intensities. Moreover, when the crystals were rotated 90° based on the (100) plane, the reflected light intensity was minimized at a different location, and the reflective light intensity of the first point was compared with the rotating crystal.

Once the intensity data for the p-polarized reflected light were obtained using the fabricated birefringence measurement system, the n_o_ and n_e_ values of the crystals were derived using Equation (1) [27]. To derive the refractive index of the crystals, the refractive index of the air medium was assumed to be one, as it was slightly different than that of air (depending on temperature and pressure) [28]. Regression of the data was conducted in MATLAB to determine the Brewster angle and calculate the refractive index. The polynomial and Fourier curve regression fittings were compared. First, we used the Fourier curve fitting and compared the result according to the number of terms used in the Fourier curve fitting. 

**Figure 6:**
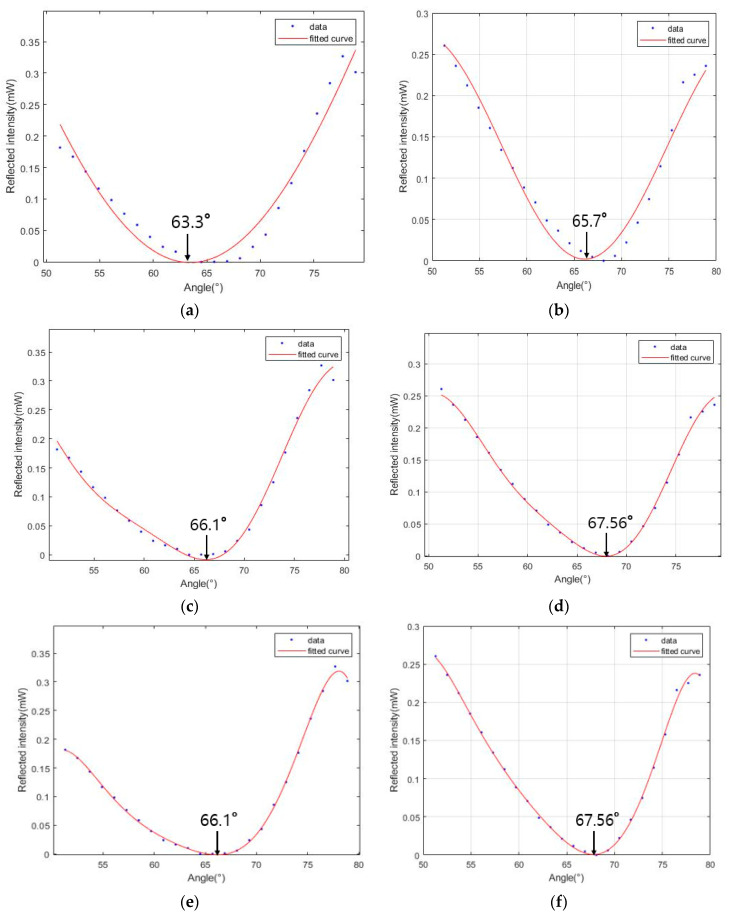

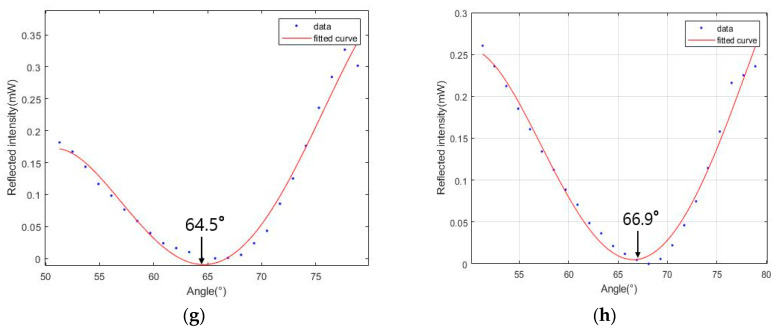
Results of the TeO_2_ crystal light intensity measurements using the developed measurement system. (**a**) Reflected light intensity related to n_o_ and the Fourier curve fitting with one term. (**b**) Reflected light intensity related to n_e_ and the Fourier curve fitting with one term. (**c**) Reflected light intensity related to n_o_ and the Fourier curve fitting with two terms. (**d**) Reflected light intensity related to n_e_ and the Fourier curve fitting with two terms. (**e**) Reflected light intensity related to n_o_ and the Fourier curve fitting with three terms. (**f**) Reflected light intensity related to n_e_ and the Fourier curve fitting with three terms. (**g**) Reflected light intensity related n_o_ and the polynomial curve fitting based on a 9th order. (**h**) Reflected light intensity related n_e_ and the polynomial curve fitting based on a 9th order.

With regard to the TeO_2_ crystal, the Fourier curve fitting was executed with one, two, and three terms. The results of the Fourier curve fitting with one, two, three, and eight terms yielded n_o_ = 1.9883, n_e_ = 2.2148, n_o_ = 2.2566, and n_e_ = 2.4213, respectively. The result of the curve fitting based on a ninth-order polynomial was n_o_ = 2.0965 and n_e_ = 2.3445, as shown in Figure 6. It was shown that the Fourier curve fitting was converged at n_o_ = 2.2566 and n_e_ = 2.4213, as shown in Figure 6c–f by increasing the number of terms, and it was more accurate as the polynomial curve fitting, see Figure 6g,h. The results of the Brewster angle, refractive index, and birefringence are shown in Table 1. Each Brewster angle error rate is 0.04 and 0.11%. The result of each refractive index error rate is 0.14% and 0.38%, respectively, resulting in an 8.21% error rate in the birefringence. 

**Figure 7:**
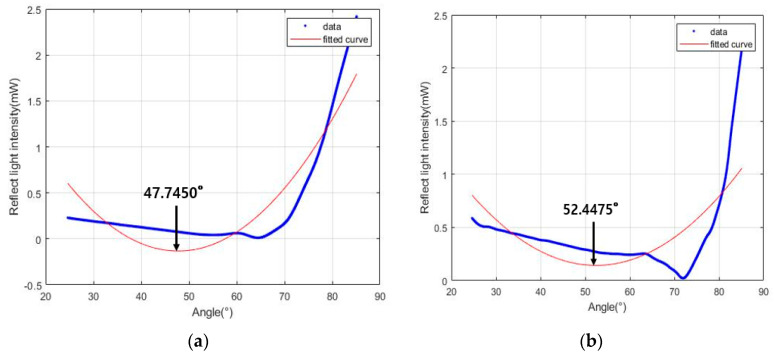

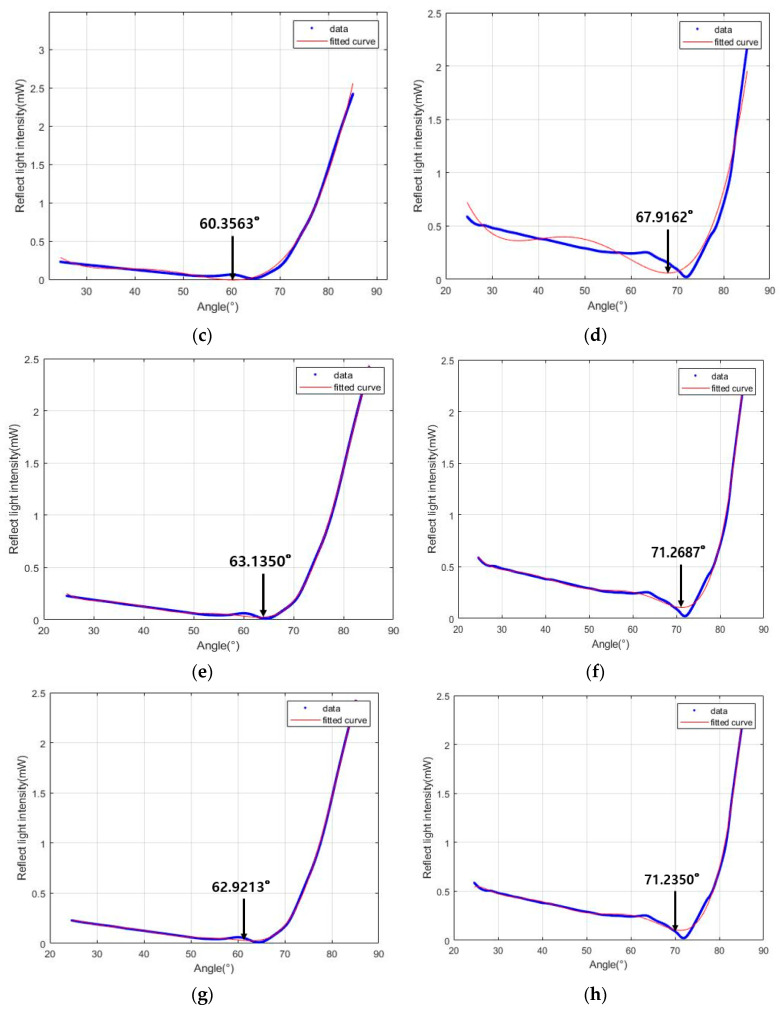

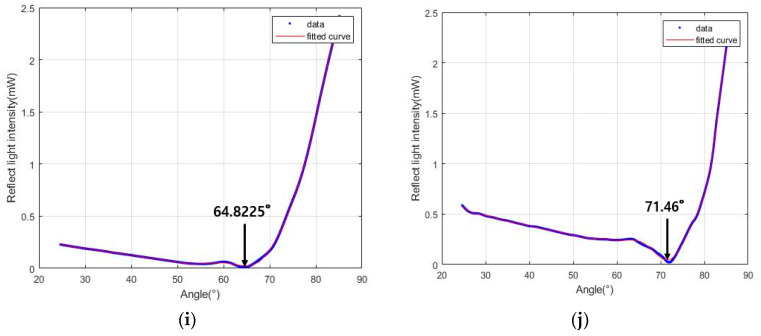
Results of the Hg_2_Br_2_ crystal light intensity measurements using the developed measurement system. (**a**) Reflected light intensity related to n_o_ and the Fourier curve fitting with one term. (**b**) Reflected light intensity related to n_e_ and the Fourier curve fitting with one term. (**c**) Reflected light intensity related to n_o_ and the Fourier curve fitting with two terms. (**d**) Reflected light intensity related to n_e_ and the Fourier curve fitting with two terms. (**e**) Reflected light intensity related to n_o_ and the Fourier curve fitting with three terms. (**f**) Reflected light intensity related to n_e_ and the Fourier curve fitting with three terms. (**g**) Reflected light intensity related n_o_ and the polynomial curve fitting based on a 9th order. (**h**) Reflected light intensity related n_e_ and the polynomial curve fitting based on a 9th order. (**i**) Reflected light intensity related to n_o_ based on the Fourier curve fitting with eight terms. (**j**) Reflected light intensity related to n_e_ based on the Fourier curve fitting with eight terms.

For the Hg_2_Br_2_ crystal, the result of the Fourier curve fitting with one, two, and three terms yielded n_o_ = 1.1007, n_e_ = 1.3008, n_o_ = 1.7572, n_e_ = 2.4647, n_o_ = 1.9741, and n_e_ = 2.9491, respectively. It was confirmed that the Fourier curve fitting was more accurate as the number of terms increased compared with the reference (where the n_o_ of the Hg_2_Br_2_ crystal was 2.12 and the n_e_ was 2.98) [24]. Therefore, the Fourier curve fitting with eight terms is executed and shown in Figure 7g, and the result of the curve fitting based on a ninth-order polynomial are shown in Figure 7h. The ninth-order polynomial curve fitting yielded n_o_ = 1.9560 and n_e_ = 2.9434 and the Fourier curve fitting with eight terms yielded n_o_ = 2.1273 and n_e_ = 2.9818. These results of the Hg_2_Br_2_ crystal show that the eight-term Fourier curve fitting has a smaller error rate of refractive indices than the polynomial curve fitting. The errors of all of the factors pertaining to the eight-term Fourier curve fitting are listed in Table 1; as indicated, the error rate was 0.12 and 0.014% for the Brewster angle, 0.14 and 0.38% for the refractive index, and 0.64% for the birefringence, respectively.

## 4. Discussion

In this study, the polynomial and Fourier curve fittings were investigated to estimate the Brewster angle using reflected light intensity data. The reflective coefficient derived by reflected light intensity can be defined as the Fresnel equation’s (including the refractive indices) combined sine and cosine functions [29,30]. Therefore, reflected light intensity is a periodical function because the incidence angle is not over 90°. For this reason, the Fourier curve fitting was adaptable in the regression of the reflective intensity. Additionally, the developed measurement system showed that it was possible to measure the Brewster angle, which is related to the refractive index. Consequently, the Brewster angles of the Hg_2_Br_2_ crystal were acquired using Equation (1) and the birefringence index of the crystal. Furthermore, their error rates could be calculated and compared using the reference data in Table 2 [31]. 

Additionally, the motor resolution was 10^−5^ (which is equivalent to a rotation of 0.01125° for a single-pulse rotation), which can be attributed to the refractive indices and birefringence resolution compared with the Hg_2_Br_2_ crystal reference data. A refractive index difference of ~0.001 occurs when the Brewster angle has an error of 0.01°. Additionally, the possibility of error was considered to have occurred during the measurement, and could be due to the change in the intensity of the laser, or the imperfect rotation angle through the motor, and the error almost perfectly matched each axis when the single crystal was cut. The errors rate of the refractive indices and birefringence in the measured Hg_2_Br_2_ crystal were less than that of LiNbO_3_ using the THz-TDS method [22]. The traditional ellipsometers use a birefringence measurement method for thin films at the millimeter scale under 10 mm; however, the developed system could measure the birefringence of single crystals at the millimeter scale over 10 mm. Furthermore, the system used is composed of low-cost mechanical devices with an optical component (He-Ne laser) for crystal irradiation. It can conduct rapid measurements using the RS-485 communication between the PC and detector module. 

Recently, the systems developed for birefringence measurement have focused on liquid crystal, thin films, and the state of residual stress [32,33]. However, AOTF requires a solid-state bulk single crystal of high quality for precise wavelengths, whereby solid-state crystals can commercialize the developed growth system. For this reason, the developed system focuses on a solid-state single crystal; accordingly, it was not possible to measure the birefringence of liquid crystal. The developed system contained an adjustable uniaxial crystal, rather than a thin film that had two refractive indices. The developed system currently has the limitation that the optic axis of a single crystal is not cut to be perpendicular. For example, the biaxial crystal requires the measurement of the three refractive indices to measure the birefringence. Nevertheless, the rotation stage used for the crystal cannot rotate about the *z*-axis in the developed measurement system. Biaxial crystals require *z*-axis rotation to match the index ellipsoid and find the optic axis. Therefore, it is difficult to measure the birefringence in biaxial crystals using the developed system. Additionally, if the single crystal has an unknown optic axis and index ellipsoid, the developed system cannot measure the birefringence. However, commercialized single crystals have well-known optic axes and index ellipsoids. Therefore, the developed system can simply be used for measuring the birefringence of well-known single crystals.

## 5. Conclusions

In measuring the birefringence, the experiment with the TeO_2_ crystal shows the range of the data collection for preventing the convergence of the results. For the Hg_2_Br_2_ crystal, the developed measurement system demonstrated a low Brewster angle (0.09% and 0.014%), refractive index (0.34% and 0.06%), and birefringence (0.64%) errors for single Hg_2_Br_2_ crystals. These results can be attributed to the rotational resolution of the motor and synchronization between the Hg_2_Br_2_ crystal incidence angle and the rotational angle of the detector. It was also demonstrated that it is possible to measure the birefringence of the single Hg_2_Br_2_ crystal when there is information about the index ellipsoid, including information related to the optical axis and cutting planes. Therefore, the developed birefringence measurement system can be used to measure uniaxial crystals in a manner similar to the measurement of the Hg_2_Br_2_ crystal. However, when a biaxial birefringent crystal that has three refractive indices is measured, the developed birefringence measurement system can consider all of the refractive indices of the biaxial crystal. Owing to the complex index ellipsoid, the developed system cannot rotate with respect to the *z*-axis of the motor. Furthermore, a single Hg_2_Br_2_ crystal that has a different planar distribution, such as (110), (001), or (−110), can be considered when the sample stage can rotate with respect to the *z*-axis. For all of the anisotropic crystal types, the developed birefringence measurement crystal should consider the rotation sample stage and the adjustable holder of the anisotropic crystal.

## Figures and Tables

**Table 1:** Brewster angle and birefringence results for the TeO_2_ crystal.

	Brewster Angle (°)		Ordinary Ray Refractive Index (n_o_)	Extraordinary Ray Refractive Index (n_e_)	Birefringence (Δn)
	Ordinary Ray	Extraordinary Ray			
Reference	66.1289	67.4806	2.2597	2.4119	0.1522
Measurement	66.1	67.56	2.2566	2.4213	0.1647
Error (%)	0.04	0.11	0.14	0.38	8.21

**Table 2:** Brewster angle and birefringence results for the Hg_2_Br_2_ crystal.

	Brewster Angle (°)		Ordinary Ray Refractive Index (n_o_)	Extraordinary Ray Refractive Index (n_e_)	Birefringence (Δn)
	Ordinary Ray	Extraordinary Ray			
Reference	64.76	71.4498	2.12	2.98	0.86
Measurement	64.8225	71.46	2.1273	2.9818	0.8548
Error (%)	0.09	0.014	0.34	0.06	0.64

## Data Availability

Data sharing is not applicable to this article.
